# Co-designing technology to improve psychological therapy for psychosis: SloMo, a blended digital therapy for fear of harm from others

**DOI:** 10.1016/j.schres.2024.11.004

**Published:** 2024-12

**Authors:** Amy Hardy, Kathryn M. Taylor, Amy Grant, Louie Christie, Lucy Walsh, Thomas Gant, Rama Gheerawo, Anna Wojdecka, Adrian Westaway, Alexa Münch, Philippa Garety, Thomas Ward

**Affiliations:** a Institute of Psychiatry, Psychology & Neuroscience, King's College London, London, United Kingdom; b South London & Maudsley NHS Foundation Trust, London, United Kingdom; c Sussex Partnership NHS Foundation Trust, Sussex, United Kingdom; d Helen Hamlyn Centre for Design, Royal College of Art, London, United Kingdom; e Special Projects Studio Limited, London, United Kingdom

**Keywords:** Inclusive design, Co-design, Lived experience, Therapy, Digital health, Paranoia, Psychosis

## Abstract

Digital technology is positioned as a potential solution to improving access, experience, and outcomes of psychological therapies for psychosis. Digital solutions need to be fit for purpose and tailored to context to deliver real world benefits. To address this, co-production is often used, where stakeholder involvement informs intervention development. However, co-production in clinical research tends to limit involvement to refining previously identified solutions to known problems. This is not an optimal approach to innovation and risks maintaining inequities. An alternative is inclusive co-design, where the needs of a diverse range of people are collaboratively explored using ethnography, and solutions to address these iteratively developed through user testing. In healthcare, we propose an evidence-based approach to co-design (‘hybrid waterfall-agile’) is required. This is because ‘agile’ exploration of needs and solutions is necessarily constrained by clinical guidelines and regulatory requirements (the ‘waterfall’). This paper provides an overview of evidence-based co-design. We use the example of SloMo, a blended digital therapy for paranoia. We describe our transdisciplinary team collaboration and how this facilitates inclusive lived experience involvement. Our therapy development method is outlined, illustrated by reflections from lived experience team members. Iterative divergent (‘zooming out’) and convergent (‘honing in’) cycles are used to co-design therapy functionality, aesthetics, interactions, and content, supported by stakeholder engagement. We conclude by reflecting on common challenges including sustaining lived experience involvement, adherence to evidence base, regulatory compliance, funding, and project management. Recommendations for navigating these obstacles are provided, with the aim of encouraging innovation in mental healthcare for psychosis.

## 1 Background

Digital technology is positioned as a solution to improving psychological therapies for psychosis. However, digital solutions need to be fit for purpose. Health inequalities may be magnified by technology (Bond et al., 2023). People with psychosis, particularly those from marginalised communities, can experience barriers to technology access and digital literacy (Aref-Adib et al., 2019). Digital therapies could also be experienced as disempowering or even coercive if they are not sensitively designed to promote agency and choice (Martinez-Martin, 2024). Inclusive, human-centred design is an approach where the needs of a diverse range of people are explored, and designs to address these iteratively developed through user testing (Clarkson & Coleman, 2015; Gheerawo, 2024; ISO, 2010; Norman, 2023). Co-design is an application of inclusive, human-centred design that involves working *with* people using ethnography (e.g., observations and interviews). But for researchers and clinicians it can be challenging to know how to go about using co-design in digital mental healthcare. The design discipline has principles and techniques which can be different to those conventionally used when developing interventions in clinical research. To add to the confusion, the term co-design is sometimes used interchangeably with co-production. However, it is important to recognise that co-production is not co-design. Co-production engages stakeholders in the implementation of a previously agreed solution to a previously agreed problem to achieve better outcomes (Vargas et al., 2022). Whereas in co-design, it is the stakeholder engagement that leads to the identification of problems and development of solutions. Previous reviews of digital mental health development have focused on evaluating co-production in design and involvement of professional designers, highlighting the need for improvement in both (Brotherdale et al., 2024; Vial et al., 2022). However, to the best of our knowledge, there is little guidance on how to employ co-design in the development of digital therapies for psychosis. The aim of this paper is to provide an overview of our evidence-based co-design approach and recommendations for addressing common challenges.

**Fig. 1:**
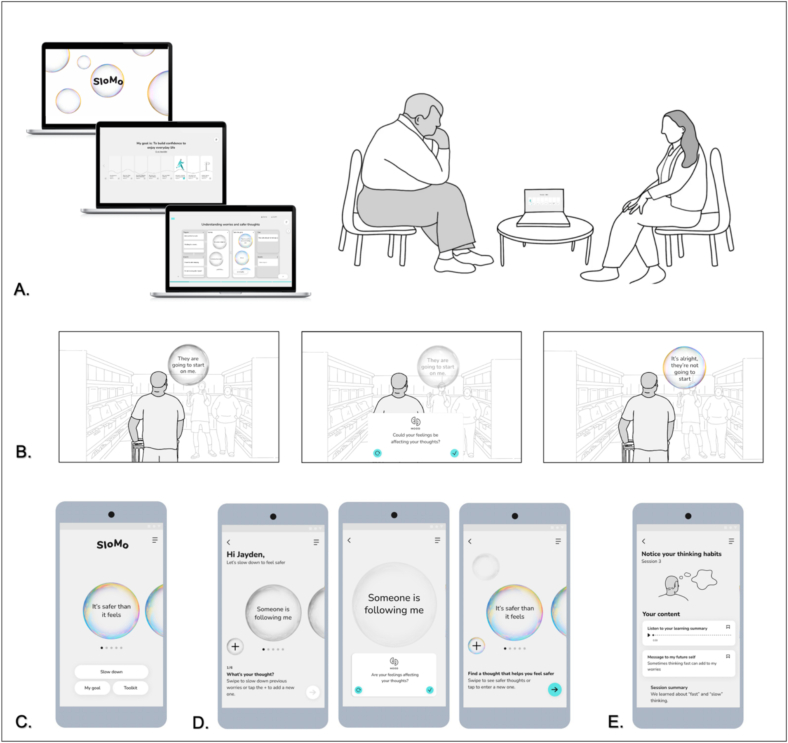
SloMo sessions with a therapist (in person or online) are supported by an (A.) online therapy platform which promotes (B.) understanding and managing worries through lived experience stories and interactive tasks. Personalised thought bubbles are visualised and synchronised with the (C.) SloMo mobile app (D.) then “slowed down” to find ways of feeling safer. Additional mobile app features can be accessed via a toolkit which includes (E.) personalised learning summaries of therapy sessions.

We will draw on the example of SloMo, a digitally supported therapy for paranoia in psychosis which is a next generation cognitive-behavioural therapy for psychosis (CBTp) (Hardy et al., 2018). It aims to improve access, experience, and outcomes of psychological intervention for psychosis. SloMo works by targeting fast thinking habits that fuel worries and supports people to slow these down to find ways of feeling safer and living well (Ward and Garety, 2019; Ward et al., 2022). It consists of a therapy platform that assists the delivery of synchronous face to face or remote sessions with a therapist, which is augmented with a mobile app for use in daily life to assist self-management (see Fig. 1). SloMo uses technology to support visualisation of thoughts and thinking habits. People interact with their personalised SloMo thought bubbles, altering speed and size to reflect related thinking habits and distress. This visual design concept transforms cognitive therapy by providing an appealing and tangible means of communicating subjective experience, whilst reducing information processing demands. SloMo tips support people to slow down and notice new information, helping to shrink fast-spinning grey, worry bubbles and grow colourful, slow-spinning helpful thoughts to promote wellbeing.

SloMo is the product of over a decade of inclusive, human-centred design by our transdisciplinary team (Wojdecka et al., 2021) of people with lived experience, designers, therapists, trainers, researchers, regulatory consultants, and software developers. The initial version of SloMo^1^ is the first UKCA/CE marked digital therapy for paranoia. It showed sustained effects on paranoia, mediated by slow thinking, over 6 months in a large multisite randomised controlled trial (RCT), alongside sustained benefits for confidence, wellbeing, and quality of life (Garety et al., 2021). Importantly, SloMo had high rates of engagement and strong user experience, which were not affected by digital literacy, age, or ethnicity, indicating that the therapy design worked as intended to support inclusion (Greenwood et al., 2022; Hardy et al., 2022). SloMo has received a NICE (2024) Early Value Assessment recommendation for use in the NHS whilst real world data is collected to address evidence gaps. Accordingly, we have developed a new, second version of SloMo, optimised for implementation based on the trial process evaluation and further co-design work. This is now being tested in a type II hybrid implementation-effectiveness study in three geographically diverse trusts in the U.K National Health Service (ClinicalTrials.gov identifier: NCT06568081).

## 2 The co-design team

A key aspect of co-design projects is creating a team that has the necessary range of expertise and perspectives. For projects based in clinical academia, this may involve collaboration across academic departments, or partnerships with industry, to bring in designers, developers, and regulatory specialists. These partnerships can bring opportunities and obstacles, as organisations may have different ways of working, for example in terms of timescales for conducting work and how it is funded. Teams need a range of lived experience expertise if working in line with best practice recommendations for co-production and co-design. This means having salaried lived experience staff who are equitably positioned in relation to other team members, as well as access to lived experience expertise outside of the research team to provide independent input. Ideally, projects should be based in ‘living labs’, whereby lived experience, clinical, technological, industry, regulatory and research expertise is brought together in an organisational structure, with co-production underpinning the co-design of digital health (Mangyoku et al., 2014). However, funding and sustaining this type of team is challenging and there are currently few examples in mental healthcare, with notable exceptions such as Orygen Digital for youth mental health in Australia.

In the case of SloMo, the work was initiated by a team of clinician academics (with AH, TW, and PG as co-founders) who, through their clinical experience, recognised the significant challenges faced by Cognitive Behavioural Therapy for psychosis (CBTp) in improving access, experience, and outcomes. Technology provided a potential way of addressing these challenges, although in the early stages of engaging with software developers it became apparent that a common approach was to simply digitise the pen and paper materials used in therapy. The co-founders were motivated to go beyond this to explore how technology could not just replicate but improve the therapy experience, through addressing common barriers to uptake, engagement, and adherence. But we lacked design expertise. This led to a collaboration with the Healthcare Design lab, Helen Hamlyn Centre for Design, Royal College of Art (RG and AWo), and subsequently a design studio led by alumni of the centre, Special Projects (AW and AM).

The Helen Hamlyn Centre for Design are pioneers of inclusive design, which is a human-centred approach that aims to make designs work for the widest number of people, and not exclude them based on aspects of their identity (e.g., age, race, ethnicity, and gender). It does this by involving people in the design process whose voices may traditionally be neglected. The SloMo co-founders strongly connected to the principles of inclusive, human-centred design. Cognitive-behavioural therapy materials have been developed predominantly by white academic clinicians based in western institutions, who are not representative of the target population of people with psychosis. Indeed, it was apparent in their clinical work that commonly used therapy tools could be unsuitable due to lacking appeal, memorability, user friendliness or being too complex to understand and apply to problems. In addition to the designers, the co-founders contracted a software company (Evolyst for version one of ‘SlowMo’, and Bitjam for version two of ‘SloMo’) to lead on coding the designs into a developed product. To support effective co-design, developers were selected based on shared values of working for the public good, a commitment to human-centred software design and meeting MedTech regulation standards.

The involvement of people with lived experience in SloMo's development has evolved over time and the team now has sufficient funding and resource to support representation at four levels, facilitating the co-design work. This includes 1) lived experience research leadership (LW), 2) expert Patient and Public Involvement (PPI) consultants with relevant expertise (in psychosis research and/or human-computer interaction, AG and LC) who are collaborators in co-design workshops and co-produced user testing sessions, 3) a Lived Experience Advisory Panel who have received training about SloMo and meet regularly to support therapy (and research) development, and 4) a pool of independent PPI consultants who have no allegiance to the therapy or team, and are involved for individual user testing sessions to test design prototypes. Crucially, inclusive design encourages purposive sampling of people with lived experience, targeting individual characteristics particularly relevant to the product, service or policy being designed. For SloMo, this included recruiting the LEAP group and PPI consultant pool to be representative of the demographics of our local populations. To support inclusive user testing, we have particularly focused on recruiting people at the tails of a normal distribution for characteristics that are relevant to therapy design. This includes people who have higher and lower motivation to engage in therapy, paranoia severity, and cognitive difficulties. The assumption in inclusive design is that if the co-design work is conducted with people who represent the “extremes” of a characteristic in a normal distribution, then the insights and solutions generated will be more likely to be suitable across the continuum.

## 3 The co-design process

**Fig. 2:**
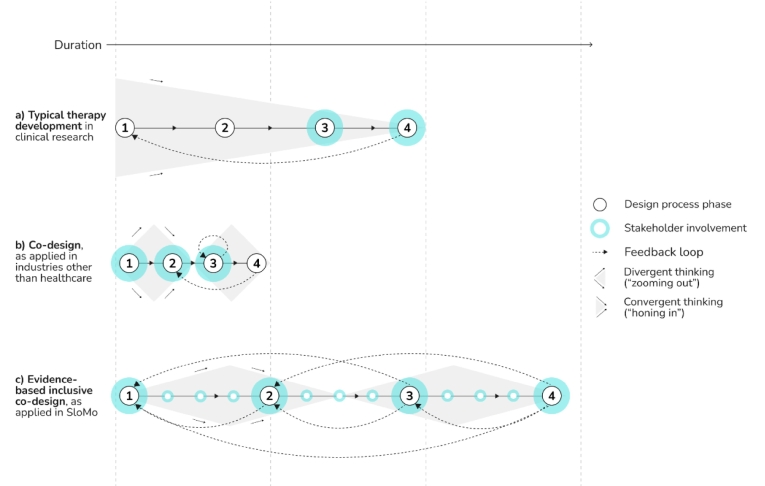
Typical processes of (a) therapy development in clinical research, (b) co-design outside of healthcare, and (c) the evidence-based co-design as employed in SloMo. See Table 1 for further detail on the functions and features of phases 1–4. (a) Convergent design process with waterfall methodology. Co-production during phases 3–4. Further development requires repetition of full cycle. (b) Double Diamond — divergent and convergent design process. Co-design during phases 1–3. Further agile development during phase 3. (c) A hybrid waterfall-agile approach combining (a) and (b): Double Diamond, constrained in divergence by the evidence base and regulatory requirements. Repeated co-design during phases 1–4. Iterative development during all phases.

To illustrate the co-design process, Fig. 2 contrasts the typical processes of therapy development in clinical research and co-design, and shows the process by which we integrated them to develop SloMo. This is complemented by Table 1 which details the functions of the phases in each design process and their key features. We recognise these represent simplifications, and that in practice, digital therapies may use a blend, tailored to development phase, research priorities and project resources.

**Table 1:** Function and features of therapy design phases in clinical research, co-design, and evidence-based co-design.

Design context	Design process phase	Function of phase	Approach	Team involvement		
				Clinical academics	Designers/industry team	PPI
Therapy development in clinical research	1	*Target the problem*. Causal mechanism(s) of a mental health problem are identified, informed by evidence base.	Waterfall	✓		
	2	*Therapy development*. Therapy techniques to modify mechanisms selected, and therapy content developed. User interaction with software and hardware relatively less of a focus.		✓		
	3	*Therapy refinement*. Stakeholder feedback through opportunistically recruited focus groups on intervention prototypes, changes made based on user wants.		✓		✓
	4	*Therapy evaluation*. Intervention is evaluated in a research study. Stakeholders involved through study participation provide acceptability data, which informs future clinical research.		✓		✓
Co-design, as can be applied in industries other than healthcare	1	*Discover the problem*. Ethnographic research conducted to understand stakeholder experiences and contexts, related to the problem, to identify *needs.*	Agile		✓	✓
	2	*Define the solution*. User needs are synthesised to provide insights into opportunities for improving experience, which may include re-framing the problem.			✓	✓
	3	*Develop solutions*. Collaborative generation of multiple possible design solutions, focusing on *both* content and user interaction with hardware and software. Iteratively tested by purposively recruited users, with changes made based on feedback.			✓	✓
	4	*Deliver a solution*. Optimal solution is selected, and a higher fidelity prototype developed for market launch. Technical validation informs future design changes.			✓	
Evidence-based co-design, as used in SloMo	1	*Evidence-based discovery of the problem*. Ethnographic research conducted to understand stakeholder experiences and contexts, related to the problem, to identify needs. Discovery limited to understanding contexts relevant to targeting identified causal mechanism(s) of a mental health problem, informed by evidence base.	Waterfall-agile hybrid	✓	✓	✓
	2	*Define an evidence-based solution*. User needs are collaboratively synthesised with stakeholders, to provide insights into opportunities for improving experience of therapy content and techniques, which may include re-framing the problem.		✓	✓	✓
	3	*Develop solutions*. Collaborative generation of multiple possible design solutions, focusing on both content and user interaction with hardware and software. Iteratively tested by purposively recruited users.		✓	✓	✓
	4	*Deliver a solution*. Optimal solution is selected, a higher fidelity prototype developed, and pre-clinical usability testing conducted with stakeholders. User feedback informs future design changes and research.		✓	✓	✓

In the clinical research process a convergent, ‘waterfall’ approach is often used, with each phase of development determined by the outputs of the preceding one to ensure rigor and fidelity. A limitation of this approach is that novel opportunities for improving user experience can be missed, and the inherent problems of the status quo are maintained. To address this risk, a key principle in co-design is to engage in cycles that are both divergent (e.g., exploring the problem to understand stakeholder perspectives or developing a range of possible solutions as prototypes) and convergent (e.g., synthesising insights from stakeholder perspectives to reframe the design problem or refining the development of the selected design solution). Co-design also conventionally adopts the agile working principle of ‘failing fast’, launching a ‘minimal viable product’ and learning from what does not work, which can expedite digital products and services being launched. However, fully agile methods can be problematic in healthcare, given the need for technologies to meet safety, technical performance, and clinical effectiveness standards prior to clinical use, in line with existing evidence and clinical guidelines.

Therefore, in developing SloMo, we have adopted a co-design process (the U.K Design Council's Double Diamond method, Design Council, 2019) and combined with evidence-based principles of clinical research, in a ‘hybrid waterfall-agile’ approach (Plant, Personal communication). The Double Diamond incorporates divergent and convergent phases, although we have adapted by constraining divergence according to the preceding evidence base. This ensures sufficient rigor in therapy development, facilitating transition of developed therapies into clinical research or implementation. Alongside this, stakeholder involvement has been critical throughout the co-design work, with iterative cycles based on user feedback, to increase the likelihood of the therapy being fit for purpose in healthcare systems. To date, we have worked through two co-design processes, the first developed version one for our RCT whereas the second optimised version two for implementation in routine care. This highlights that there is no definitive ‘end-point’ in the co-design of digital therapies, as they can continue to be improved based on learning and new use cases. Below we elaborate on our co-design process, illustrated with reflections from our expert lived experience team members (AG and LC).

“I see the options for developing therapies as being like kinds of train journeys: the clinical research train has one route, with just a few stops to pick up passengers near the end. Passengers can only influence that journey, like whether to sit or stand. In co-design, the train could take any route, to any destination, dependent on where passengers need to go. But once a destination is decided, its fixed. When developing SloMo, we knew we had to follow certain routes, but we picked up lots of passengers over multiple trips to find out what worked best. It took longer overall but hopefully in the future it means that more passengers will have a good experience of their journey and get to where they want to go.”

AG

### 3.1 Design inputs: discovering and defining opportunities for solving problems

The early phases of design require adopting a curious stance to broadly explore the problem. In contrast to therapy development in clinical research, this means not just identifying the therapeutic targets and techniques that will modify them based on existing evidence. Instead, a range of stakeholder views and perspectives should be iteratively investigated using ethnographic methods.

“SloMo is for people like me so I felt like I was there to see what worked and what didn't. And why this worked and why that didn't work. So we can do more of this and less of that. My presence felt equal with everyone else. I felt I had a specific purpose and that everything I shared was valid. I could voice my opinion; I've been involved in previous projects where I did not share my lived experience. I felt like I'd been shut down, but I didn't feel like this in this project. I felt comfortable enough to talk, and that's the most important thing in any meeting; otherwise, nothing will change; that's the basic requirement: someone must feel safe enough to talk. Otherwise, mental health will miss out on new ideas and innovative perspectives.”

AG

Another key distinction with clinical research is that co-design investigates what people *need,* instead of what they say they *want*. A common approach in clinical research projects is to decide therapeutic targets, then convene focus groups to ask people what they want an intervention to include. This is not co-design (Norman, 2023). Instead, a range of ethnographic research methods are used to understand people and their contexts. Co-design activities can uncover unanticipated needs that emerge through conversation, confusion, observing behaviour, and body language. They are also valuable to build a shared language that can then be used in the design, which is particularly helpful for designers who are not as familiar with the field.

“I approached everything from two different angles, my experience of computer programming/developing apps, as well as my lived experience. I had empathy for the user, and technical insights into what's possible. I was able to contribute many small improvements to the co-design. Particularly tweaking of the language used. Alongside other PPI advisors, using our lived experience of paranoid thoughts, we removed a lot of “psychologists' jargon and made the language less triggering.”

LC

The understandings derived from this divergent work are then used to formulate a definition of what *needs* the design solution should meet (or the ‘value proposition’) which often involves ‘reframing’ the initial problem. Note these *needs* may not be a direct reflection of what people say they *want*, if addressing *wants* means *needs* are unaddressed or even exacerbated. For example, a person may understandably say they *want* as much therapeutic content as possible available in a digital therapy to help them manage their anxiety. However, interviews and observations may reveal that this is overwhelming and what they *need* is to be able to easily access their most helpful strategies. The reformulation of problems into needs or opportunities is a fundamental aspect of co-design that can identify novel possibilities for improving healthcare, in contrast to how therapies are conventionally developed.

When SloMo was initially developed for our RCT, the ethnographic research included observations of therapy sessions and mental health team meetings, and interviews with a wide range of service users, therapists, and trainers. From this, we developed inclusive user profiles (personas of typical users) and mapped out user journeys to reflect the diversity of users and their contexts. This was complemented with desk research into gaming, technology use in healthcare, and the representation of thoughts and thinking habits in the arts. Insights from this work led to the design solution being defined as a therapy that went beyond the initial aim of improving outcomes by targeting thinking habits, to also address broader needs by supporting monitoring, simplifying information processing, enhancing enjoyment and trust, promoting personalisation and normalisation, and offering flexible interpersonal support. When we conducted the second co-design process, the initial phase used feedback from participants and therapists in the RCT to identify further possibilities for content, functions, and interface in relation to the original design definition. Interdisciplinary co-design workshops were then held to further explore mental models of paranoia and therapy, interface options for the therapy, and identify common concerns or dilemmas that the therapy design needed to address.

### 3.2 Design outputs: developing and delivering the design solution

Once an understanding has been developed of the design needs, the next stage focuses on creating therapy prototypes and iteratively testing and refining them based on feedback from end users (patients, carers, therapists, and trainers). Prototypes are mock-ups or representations of the therapy look and feel, content, or interface interactions. As the beginning of this phase involves divergently exploring possible solutions, the aim here is not to code the final product, but to develop basic materials (in pen and paper, or digital format) to explore potential options. From a design perspective, it is recommended to test the prototype in the context it will be delivered, so for individual therapies the user testing should be conducted with individual or pairs of users (therapist and/or patient). User testing focuses on observing non-verbal behaviour and reactions, and eliciting verbal feedback on experience, attitudes, and expectations. Sampling of individuals for user testing and feedback elicited should be tailored according to the specific aims of the testing session. For example, when testing interface interactions, it is important to ensure testing is conducted with people who lack skills or confidence in using technology, to ensure that selected designs meet their needs.

“It's creating a ‘positive algorithm’ of what works and is preferred. This takes a lot of the guesswork out of ‘I think this will be helpful’ vs actually demonstrating the idea. Which to me feels essential to keeping a product relevant, when you're actually asking the target audience, ‘do you find this helpful?’.”

AG

“User testing was the most enlightening part for me. It was a great way to get empirical evidence to oppose biases. For example, we did user testing of three different versions of the animation style, with people from BAME backgrounds, who are disproportionately affected with mental health issues, and their input helped to shape the design. This is important because psychologists, tend to be affluent, white women. Designers also have similar backgrounds. Testers told us it was important that everyone was represented: different races, different waistlines, braided hairstyles.”

LC

When a final design solution is selected, a higher fidelity prototype is then iteratively developed through usability testing, with the aim of an initial validation of user experience (i.e., usefulness, ease of use and satisfaction). Once established, the final prototype can then be taken forward for efficacy, effectiveness, and implementation evaluation in clinical research, or rolled out in routine care once clinical benefit and positive user experience have been demonstrated.

For both versions of SloMo therapy (i.e., the version tested in the RCT and the subsequent version that has been optimised for implementation) iterative cycles of development testing were conducted. Initially these focused on the concepts underpinning the therapy, and aesthetics, then moved into testing how people interacted with basic digital prototypes. Wireframes of the therapy platform and mobile app (i.e., diagrams detailing the screen content, functionality, and architecture) were reviewed in co-design workshops and used to identify dilemmas and concerns. These were resolved through user testing specific design problems (e.g., what attributes are needed for a personalised avatar and how do people experience different ways of interacting with thought bubbles). This approach allowed us to turn the co-design outcomes into ideas that evolved over time until they met the user needs and were ready for testing in clinical research. Table 2 provides reflections from our expert PPI consultants on the second co-design process, highlighting how SloMo was further optimised to meet the identified needs of a psychological therapy for paranoia.

**Table 2:** PPI reflections on using co-design to optimise SloMo for implementation.

Needs for SloMo therapy	Quote
Supporting monitoring	“*We prototyped different slider mechanisms for rating how worrying thoughts were. Both with and without numbers, and with different scales, and interactions. We tried them out with people to find which was most user friendly. I do remember the most popular was unexpected and could not have been predicted any other way than user testing. We ended up with a simple slider interaction and scale, on the second or third iteration, based on the user feedback.*” (LC). “*The common denominator was people saying in different ways that, ‘they find talking about depressing things depressing’ and ‘It felt good to talk about this or that positive aspect.’ So if people are responding positively to focusing on the positives they are able to achieve, it's good to build on that* vs *starting from ground zero where there isn't a seed of hope. It's easier to plan goals when you can see proof that growth is possible. Whereas, reflecting on the negatives can prevent knowing that the things you want in life may not be impossible. A positive perception might not enable you to climb a mountain but it can help get you to show up and try.*” AG
Simplifying information processing	“*Observing user behaviour informed the design of controls of the app. I'd assumed that everyone knows how to ‘pinch-to-zoom’ on a mobile phone from using Google maps, and soon learned that most people are not as computer literate as myself - we needed a simpler mechanism.*” LC “*Not many people can deal with reading a lot of information, and some people process information differently. It was important to me that the app and website shared information in different creative ways. Which is why I love the vignettes and the therapy really resonated with me. I think the audio stories are easy to follow along and impactful because it feels more relatable.*” AG
Enhance enjoyment	“*I suggested that the last therapy session in the therapy platform should include a celebration, like confetti. Something positive that the person could see when they got to the end of therapy. The designer jumped up there and then and added it to the app.*” AG “*Therapy can feel uncomfortable, but it also needs to feel safe and a tolerable level of discomfort; the balance has to be correct. I felt like SloMo is a different approach to therapy; it feels more focused on the positives and highlighting the positives instead of the usual way where you work on your problems and mainly discuss the negatives; I know when we even changed the landing page to focus on someone's positive thoughts, things like this are important. Adding more positive influences and prompts so people want to stay engaged and continue with SloMo.*” AG
Enhance trust	“*I remember the designers had included a feature where a therapist could double tap on an item to take a secret private note about it for later. I raised how this would not work as when I was at my most unwell I was hypervigilant. Someone with paranoid thoughts would notice, and feel spied on. It was agreed that, as a general rule, everything needed to be transparent to the service user, and the secret note feature was removed.*” LC “*When designing the app, there was a feature where something someone added would be automatically shared with the therapist. I highlighted how someone may not want everything they put on the app to be shared; they might want to keep it private. Someone might not like everything they have shared to be saved to their file. I wouldn't. If you download an app, you want it to be for you. The feedback I shared about this helped shape how the app was built.*” AG
Promote personalisation	“*Throughout the project I was adamant that everything was about choice, the person's choice. It is more powerful that way. If someone feels they have some control and power over their choices, I hope they will feel more comfortable about having digital therapy.*” AG “*One size does not fit all. When giving my feedback, I tried to make everything feel more personalised, not necessarily to me, but to the person using it. Someone must know about what they are using and what they are getting into, especially for someone who might be experiencing paranoia.*” AG
Promote normalisation	“*I feel like we contributed a lot to the design around the voice, language and tone used throughout the app. We made it warmer, so it felt less intimidating and more comfortable for people to use. Contributions like this make a big difference for someone using the app.*” AG
Offer flexible interpersonal support	“*I enjoyed the DIY approach SloMo provides. You can do sessions by yourself outside of the therapy room and put what you have learned into practice. I was able to access the knowledge of the sessions, without needing to keep reading it over and over. I see the “community tool” as having potential to develop into a digital journal that would be an excellent resource enriched by people sharing valuable information, personal observations, stories, ideas, different voices, anecdotes and all the stuff in-between, for future reference. It will help create a database that can be used for self-reflection, inspiration, motivation, intellectual exploration and creativity.*” AG

## 4 Co-designing digital therapies for psychosis: challenges and recommendations

Employing evidence-based co-design in therapy development is more time consuming and complex compared to conventional clinical research. It is therefore common to encounter obstacles in this work. Reflecting on SloMo's development, we have identified ways we could have improved our co-design practices and processes to better support the team and our outputs. Accordingly, Table 3 outlines common challenges, together with recommendations for how to mitigate their impact on project progress and outputs. We hope that these will provide useful guidance for other clinical academic transdisciplinary teams working in the field, even when it is not possible to comprehensively adopt an evidence-based co-design approach. Most importantly, we strongly recommend that people's lived experience is considered throughout the development process, in relation to all relevant stakeholders. This can deliver impact not just for the project, but also for team members, creating reciprocal benefits for research and PPI.

**Table 3:** Challenges and recommendations for evidence-based co-design of digital therapies for psychosis.

Challenge	Description	Recommendation
Ensuring sufficient diversity in involvement	• Engagement with seldom-heard groups can be challenging, resulting in PPI that is not sufficiently representative of the target populations.	• Relationships matter. Plan and invest time and resource in building and maintaining connections with relevant individuals and their networks (e.g. people with lived experience, carers, trainers, therapists). • Where possible, develop ‘living labs’ of stakeholders within an organisation, to provide infrastructure to identify, involve, reimburse, support, and develop people involved in therapy development projects. • Recruit people that represent a wide range of backgrounds and attributes, relevant to the therapy development and target populations, and purposively sample them according to the aims of specific user tests.
Adherence to evidence base, MedTech and research regulations	• Constraints on how much design work can diverge to explore novel possibilities, as therapy development needs to adhere to empirical evidence and clinical guidelines. • Medical device regulation standards need to be followed when making design changes to certified devices or devices under notified investigations, which can mean that they require notified investigations or amendments prior to being implemented, requiring additional time and cost.	• Be transparent at the start about what is not possible to change. Balance with creating a space where all ideas are valued, even if not feasible for the therapy or at this stage. Be cautious of inadvertently shutting down creativity and divergent thinking that provides insights as to people's needs and helpful design ideas. • Recognise that divergence may reduce as therapies progress to adoption in healthcare, as the design will have been iterated to be fit for purpose. Although divergence may still facilitate repurposing of therapy to new use cases. • Ensure thorough co-design work and pre-clinical usability testing occurs before clinical research, when it is more difficult to make changes.
Project management	• Communication can be difficult when people have differing expertise and roles on the co-design project (including time commitment, background experience, usual working practices, investment in project delivery). • People with lived experience may understandably have personal circumstances that impact availability which can impact progress and continuity. • Can be difficult to balance progressing with new tasks whilst also ensuring sufficient time to review progress to date and background, so that team members are up to date and have a shared understanding.	• Ensure key documents, outputs, and progress updates relevant to the co-design project are available to all team members, and provided in accessible formats where possible (e.g. suitable for text-to-speech readers, allowing dark/light modes, plain English summaries). Documents could include project overviews, timeline of tasks, planned outputs, outputs from ethnographic research (e.g. personas/user profiles, user journeys, system mapping) and team member contacts. • Have a system for documenting outputs of co-design activities, such as PPI workshops or user testing sessions. Record design change ideas and collaboratively agree priority ranking for development, given resources, timescales and regulatory requirements. • Provide flexible opportunities for stakeholder involvement and ask people how they want to be included. Tailor modality of sessions (face to face, video, phone, email) and duration of sessions to individual preferences, as far as possible. Acknowledge where there are constraints (e.g. having to hold workshops remotely due to geography, longer sessions due to costs or availability) and provide workarounds where possible. • Support all team members and stakeholders. Provide regular supervision for team members and opportunities for briefing and debriefing user testing sessions. • Facilitate all team members' development with opportunities for training and public engagement.
Funding & resource planning	• Traditionally funding schemes support research but not digital therapy development. • Budgets tend to be more limited in clinical academic than a commercial context, placing constraints on project outputs. • Designers and software developers may be external suppliers and relatively more costly than inhouse collaboration. • Particularly for early-stage projects, it is difficult to obtain funding when the planned output cannot be specified in detail at the stage of application.	• Explore specialist funding schemes that support product design and development (e.g., NIHR i4i) and include realistic budgets in applications (including product management, PPI, software development, design and regulation). • Prioritise design and development tasks (i.e. essential vs desirable vs roadmap (i.e., future) features) and allocate resource accordingly. • Develop low fidelity solutions where possible (e.g. pen and paper prototypes). Be cautious of investing time and resource in development that may not deliver sufficient benefit (e.g. costly animations when a simpler version may have comparable impact). • If not feasible to engage professional designers, ensure that an understanding of stakeholder experiences is embedded throughout the project and that co-design focuses on therapy interface and user experience, not just therapy content and clinical outcomes. • Sufficient detail of co-design work should be given to support the application whilst allowing for flexibility in the final outputs.

“I think overall doing co-design workshops helped me to improve my self confidence, develop my computer literacy and IT skills, and pursue my personal goals regarding advocating for mental health. It was cathartic being able to use my lived experience to help develop and add input to a project that will help others. It made me feel part of the team. What I say matters, which is one reason I have stayed involved with the project. I think being able to build my confidence regarding sharing my ideas and lived experiences. This has allowed me to see that I am capable of contributing to society in my own way that feels meaningful.”

AG

“Being a PPI advisor doing co-design on the SloMo mobile app and therapy platform was helpful for me. I consider it part of my recovery. It gave meaning to my past difficult experiences by using them to help others. The paid occupation was helpful during a global downturn and it gave me confidence to get more work so it was helpful to taxpayers and society too. I feared my name attached to an academic paper related to mental health would lose me jobs. I was surprised to discover the more I talk about my experiences, the more doors seem to open.”

LC

## 5 Conclusion

We have outlined how evidence-based co-design has been used to develop SloMo, a digitally supported therapy for psychosis, with the aim of improving access, experience, and outcomes. Our aim has been to highlight the value of going beyond just involving stakeholders to implement previously agreed solutions, as in co-production, to instead harnessing stakeholder involvement to develop solutions based on needs and tailored to context. We propose that this approach is better positioned to deliver innovation in mental healthcare that delivers real world benefits. Implementation-effectiveness evaluation of SloMo's use in routine care is underway. We plan to use the outputs from this work to continue improving SloMo, in pursuit of optimising its design for the widest possible range of people and their healthcare settings.

“You have to be actively mindful about the question, ‘Can we do more?’, to keep the engine of inspiration going and finding new ways to help and innovate.”

AG

## CRediT authorship contribution statement

**Amy Hardy:** Conceptualization, Data curation, Funding acquisition, Writing – original draft, Writing – review & editing. **Kathryn M. Taylor:** Conceptualization, Data curation, Writing – original draft, Writing – review & editing. **Amy Grant:** Conceptualization, Writing – original draft, Writing – review & editing. **Louie Christie:** Writing – original draft, Writing – review & editing. **Lucy Walsh:** Conceptualization, Writing – original draft, Writing – review & editing. **Thomas Gant:** Conceptualization, Writing – review & editing. **Rama Gheerawo:** Conceptualization, Writing – review & editing. **Anna Wojdecka:** Conceptualization, Writing – original draft, Writing – review & editing. **Adrian Westaway:** Conceptualization, Writing – review & editing. **Alexa Münch:** Conceptualization, Writing – original draft, Writing – review & editing. **Philippa Garety:** Conceptualization, Funding acquisition, Writing – review & editing. **Thomas Ward:** Conceptualization, Funding acquisition, Writing – original draft, Writing – review & editing.

## Funding sources

AH and TW acknowledge funding from the Maudsley Biomedical Research Centre at South London and Maudsley NHS Foundation Trust and 10.13039/501100000764King's College London. The authors acknowledge therapy development funding from the 10.13039/100012176Maudsley Charity, the Helen Hamlyn Trust, and PG and Professor Elizabeth Kuipers 10.13039/501100000272National Institute for Health Research (NIHR) Senior Investigator awards. The authors acknowledge funding for the SlowMo RCT from the 10.13039/501100001922Efficacy and Mechanism Evaluation Programme, a 10.13039/501100000265Medical Research Council (MRC) and NIHR partnership Project, ref. 15/48/21. The EME Programme is funded by the MRC and NIHR, with contributions from the Chief Scientist Office in Scotland, National Institute for Social Care and Health Research in Wales, and the HSC R and D Division, 10.13039/501100001626Public Health Agency, in Northern Ireland. The views expressed in this publication are those of the authors and not necessarily those of the MRC, NHS, NIHR, or the Department of Health. This work was supported by the 10.13039/100010269Wellcome Trust [109,586/Z/15/Z] and [225,816/Z/22/Z].

## Declaration of competing interest

The authors declare that they have no known competing financial interests or personal relationships that could have appeared to influence the work reported in this paper.

## Glossary

Co-production: practice of involving stakeholders to support the implementation of a previously agreed solution to a known problem.
Inclusive, human-centred design: an approach where the needs and preferences of a diverse range of people are explored, and solutions to address these gradually designed through repeated user testing.
Ethnography: a research discipline where people are investigated using qualitative methods such as interviews and observations. It aims to understand individuals and societies.
Co-design: practice of working *with* people using methods to improve the design of products, services, and policies.
Divergence (‘zooming out’): design stage where people's needs and contexts, or possible design solutions, are broadly explored to identify novel insights and opportunities.
Convergence (‘honing in’): design stage where the design task is defined or where a selected design solution is refined.
Double Diamond: visual representation of the stages of a human-centred co-design framework, including two diamonds each with divergent and convergent phases. The first diamond seeks to discover the ‘root’ problems and needs, then defines the design opportunity. The second develops possible solutions and then delivers the selected ethnography design, based on user testing.
Waterfall-agile: a *hybrid* approach to medtech development, in which a ‘waterfall’ model necessarily guides aspects of intervention development that are established in the existing evidence base, in line with regulatory requirements, while ‘agile’ methods enable flexible, iterative optimisation in response to user needs and behaviour, to facilitate adoption by stakeholders in healthcare settings.

## Footnotes

1 The first version of the therapy was ‘SlowMo’. Following stakeholder feedback and observation indicating problems with the spelling of ‘SlowMo’, further user testing indicated ‘SloMo’ was more usable and had fewer problematic connotations. The spelling was updated for version two.
